# Brain Point of Care Ultrasound in Young Children Receiving Computed Tomography in the Emergency Department: A Proof of Concept Study

**DOI:** 10.24908/pocus.v8i2.16435

**Published:** 2023-11-27

**Authors:** Stephanie R Davenport, Nadya Ben Fadel, Jorge Davila, Nick Barrowman, Vid Bijelic, Allan E Shefrin

**Affiliations:** 1 Division of Emergency Medicine, University of Ottawa Ottawa, Ontario Canada; 2 Division of Neonatology, University of Ottawa Ottawa, Ontario Canada; 3 Division of Diagnostic Imaging, University of Ottawa Ottawa, Ontario Canada; 4 Clinical Research Unit, University of Ottawa Ottawa, Ontario Canada

**Keywords:** POCUS, Pediatric, Pediatric Emergency Medicine, Intracranial Hemorrhage, Trauma

## Abstract

**Background**: Point of Care Ultrasound (POCUS) is an important tool in pediatric emergency medicine. In neonatal intensive care medicine ultrasound is often used to evaluate the brains of sick neonates. In theory, POCUS could be used in the ED in young children to evaluate the brain for abnormal pathology. **Objectives**: To examine the ability of PEM faculty to use brain POCUS to identify clinically significant brain injuries in children with head injuries and/or abnormal neurological exams, and generate sensitivity and specificity of brain POCUS in assessing such findings. **Methods**: This study used a convenience sample of patients seen in a tertiary care pediatric centre who required a CT head. A team of physicians who were trained at a workshop for brain POCUS were on call to perform the POCUS while being blinded to the results of the CT. **Results: **21 children were enrolled in the study. Five (24%) of the patients had a CT that was positive for intracranial bleeds. Of the 5 patients with a positive CT, 3 had a brain POCUS scan that was also positive. The two false negative brain POCUS scans were on patients with small bleeds (no surgical intervention required) on CT, as reported by radiology. The sensitivity of brain POCUS was 60% (CI 15% - 95%) with a specificity of 94% (CI 70%-100%). The diagnostic accuracy of brain POCUS was 86% (CI 64% - 97%). **Conclusion:** This small proof of concept study shows that brain POCUS is an imaging modality with reasonable sensitivity and specificity in identifying intracranial pathologies that are present on CT. Its use may be most beneficial to expedite definitive imaging and subspeciality involvement.

## Introduction

Over the last 15 years, point of care ultrasound (POCUS) has emerged as one of the most important and most utilized tools in pediatric emergency medicine (PEM) 1, 2. What was initially used as a screening tool in the assessment of major traumas to help determine the requirements for further investigation or management is now used in over 40 clinical applications including the assessment of intra-abdominal hemorrhage, cardiac views to assess function and fluid, the identification of testicular torsion and the evaluation of skull fractures^1^, to list a few. 

In PEM, the main modality used to urgently investigate children presenting with acute symptoms or signs consistent with brain injury and/or other pathology is unenhanced computed tomography (CT) of the head. Although CT provides clear and accurate results, it is accompanied by several downsides. First, CT uses ionizing radiation that in children has been associated with increased risk of secondary malignancy^3^. This is especially true in young children whose tissues are particularly vulnerable to radiation^3^. Second, CT can be resource-intensive, especially in the middle of the night, when CT technicians and radiologists may not be readily available in hospital, which can lead to delays. Finally, some patients need to be sedated to transfer safely to CT and to acquire high quality images, exposing these patients to the additional risks of sedation. In contrast, POCUS is readily available at the bedside without delays and many PEM physicians are already comfortable using it for a wide variety of applications 4. Physicians can repeat the assessment as often as clinically indicated as it imparts no radiation risk and requires no sedation or patient transportation. Finally, POCUS provides information immediately to the treating physicians which streamlines care, decreases length of stay, and increases patient satisfaction 1.

In neonatal intensive care, POCUS brain (described as cranial ultrasound in the neonatal literature) has become the primary imaging modality to evaluate intracranial pathology for many of the same reasons noted above 5. POCUS is preferred over other modalities like MRI (considered the gold standard) or CT, (rarely used due to radiation) because POCUS offers ease of use and portability to the bedside of often critically ill neonates for whom moving or sedating them for alternate imaging proves potentially dangerous^5^. Furthermore, it has been shown in the neonatal literature to have high sensitivity and specificity for detecting intracranial pathology when compared to post-mortem examination of the brain^5^.

The use of brain POCUS in the emergency department setting has been largely unstudied. There have been two studies published thus far in the literature that examine the use of brain POCUS as a point of care modality in the setting of trauma. The first study published by Elkunovich et al, was a retrospective study looking at brain POCUS versus CT^3^ and a second study published by McCormick et al. looked at infants under two years of age presenting with closed head trauma and positive CT findings^6^. To date, no one has attempted to see if brain POCUS can be used in the emergency department to evaluate the brain of children presenting with head trauma and/or abnormal neurological exams. 

In this pilot study, our primary aim was to examine the ability of brain POCUS to identify clinically significant brain injuries as defined by the Pediatric Emergency Care Applied Research Network (PECARN) as traumatic brain injuries that require neurosurgical intervention, intubation, hospital admission, or result in death 7. Our secondary aim was to determine the sensitivity and specificity of brain POCUS in our population of children <15mo. compared to CT. 

## Methods

### Setting/Study Population

This was a prospective feasibility study using a convenience sample of patients seen in the ED at an academic tertiary care pediatric hospital over a 2-year period. This hospital has an annual ED census of over 72,000 Patients. Over the last five years there has been an average of 30-50 children per year under the age of two who have undergone a CT of their head from the ED to evaluate for acute intracranial pathology secondary to trauma or infection. All pediatric emergency physicians who were POCUS-trained were invited to participate in a two-hour POCUS brain workshop, designed with expertise and input from a NICU staff physician (NBF), who is formally trained in ultrasound. The brain POCUS protocol and study parameters were discussed during the workshop and there was opportunity for hands-on practice on infants. The ethics review board at this hospital reviewed and approved all aspects of this study. Please seen appendix 1 for full details of POCUS training. 

### Data Collection 

All children <15 months of age presenting to the Emergency Department with head trauma or abnormal neurological signs and symptoms deemed to require a CT head by the treating ED staff physician were eligible to participate in the study. After enrollment and informed consent, each child that the treating physician sent for CT head also received a brain POCUS scan when a member of the brain POCUS team was available. The lead brain POCUS physician (SD) was on call for the department evenings and weekends most days of the month. The physicians performing the brain POCUS were blinded to the results of the CT head. The brain POCUS team physician then recorded whether they saw any abnormalities on a standardized form (Supplementary Appendix 1). This form also contained demographic information as well as other measures like the size of the fontanelle. After the results were compiled the lead author would review the CT formal reports and record any differences between the findings from the POCUS brain documentation and the formal CT radiology report (e.g., bleeds). Both the CT and brain POCUS images were then read by the radiologist on our team to ensure quality of the brain POCUS images as well as to see if additional findings on brain POCUS were missed by the performing physician. The radiologist was blinded to the results of the CT until analysis of the brain POCUS results were documented. 

### Statistical Analysis

Sensitivity and specificity were calculated using the positive and negative findings reported on brain POCUS versus those formally reported on CT. Positive and negative predictive values were obtained similarly. The diagnostic accuracy was calculated comparing the positive findings on CT and the positive findings on brain POCUS. The kappa coefficient was calculated using the positive and negative reported findings on brain POCUS by the brain POCUS physicians and compared to the findings as reported by our radiologist who interpreted the brain POCUS results. 

## Results

During the study period 21 children were enrolled. The mean age was 3 weeks and half of the patients were female. The most common indication for patients to receive a head CT was for trauma. The other indications were primarily for abnormal neurological exams. 95% of the patients were thought to have an open fontanelle by palpation, but we were able to obtain coronal and sagittal views in 100% of the patients enrolled through the anterior fontanelle window (Table 1).

**Table 1:** Participant characteristics (n=21).

**Characteristic:**	**Result:**
Age (weeks): Mean (SD)[range]	3.1 (2.8) [0.5, 13]
Sex:	11 F (52%), 10 M (48%)
Indication for CT/POCUS:	76% (16/21) Trauma 14% (3/21) Abnormal Neurological exam (e.g., Abnormal eye movement, seizure etc.) 5% (1/21) Cephalohematoma 5% (1/21) Subgaleal Hematoma
Anterior Fontanelle open by palpation:	95% (20/21)
Images obtained (%)	100%

Overall, 18 (86%) of the CTs performed in our study population had a positive finding, though most were not necessarily of clinical significance. Five patients (24%) had a positive CT that showed a skull fracture. Five (24%) of the patients had a CT that was positive for intracranial bleeds. The remainder of the positive CT findings (8/18) were for incidental findings like ethmoid opacification, asymmetrical ventricles, or prominence of the extra-axial space etc. (Table 2). Of the five patients with CT that had significant findings (intracranial bleeds), 3 had a brain POCUS scan that was also positive. Two patients had false negatives on brain POCUS where CT revealed small bleeds. The first was an extra-axial hemorrhage in the occipital area and the second had a small subdural hematoma in the parietal region. Among the study patients, there was one patient who had a false positive scan by brain POCUS for a bleed not seen on CT.

**Table 2:** Participant characteristics (n=21).

**Findings**	Number of Positives (%)
Positive CT findings overall	3.1 (2.8) [0.5, 13]
Positive CT for fracture only	11 F (52%), 10 M (48%)
Positive for bleed	95% (20/21)
Positive for incidental findings	8 (38.1) · Mild prominence of the extra-axial space in frontal regions

The sensitivity of brain POCUS using only the clinically significant positive CT scans for bleeds was 60% (95% confidence interval [CI] 15%-95%) with a specificity of 94% (CI 70%-100%). The positive predictive value and negative predictive value were 75% (CI 19%-99%) and 88% (CI 64%-99%) respectively. The diagnostic accuracy of brain POCUS was 86% (CI 64%-97%).

When the radiologist reviewed all the images obtained by the clinician there was overall agreement of 86%. There were 4 instances (19%) where the POCUS clinician was able to identify positive pathology that were not visible to the radiologist on repeat analysis of the POCUS brain images. The inter-rater reliability was 35% (CI 17%-97% p=0.05).

## Discussion

**Figure 1:**
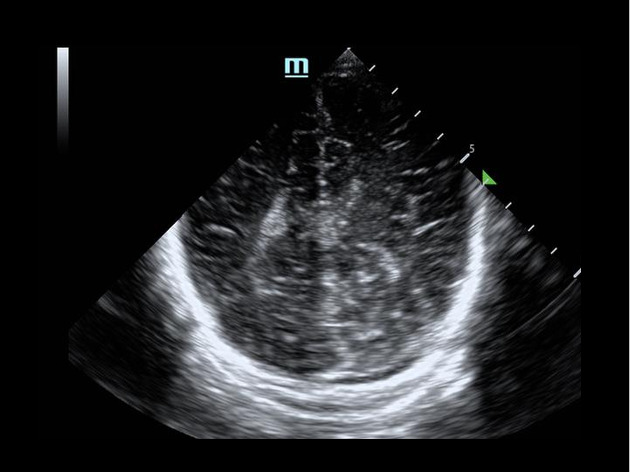
Brain POCUS of a patient with a subdural hemorrhage, coronal view.

**Figure 2:**
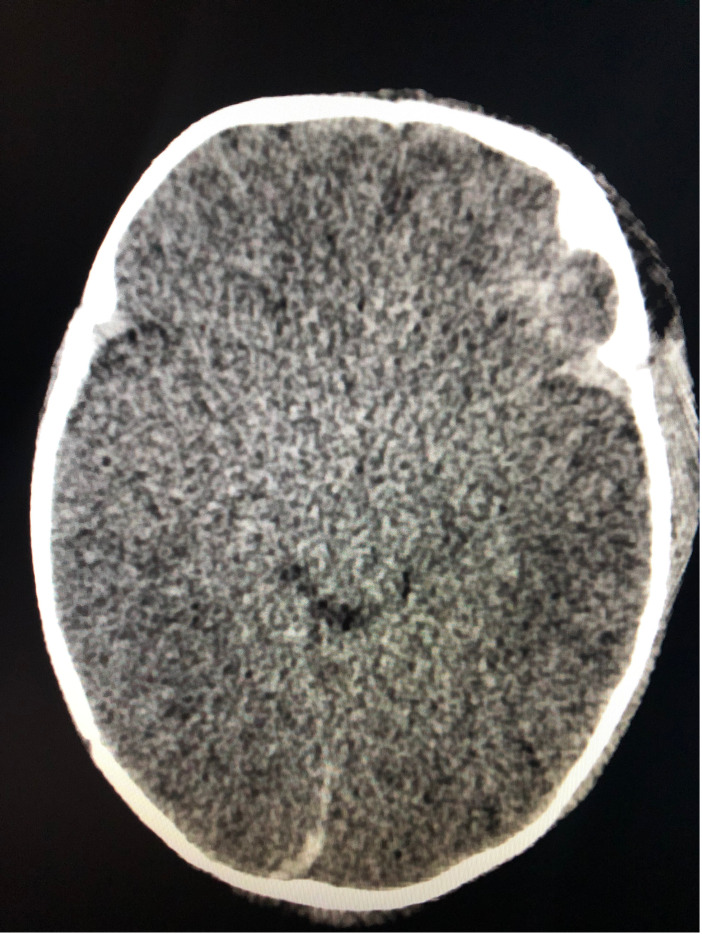
CT of the same patient with a subdural hemorrhage.

In this study we were able to demonstrate that brain POCUS can identify intracranial abnormalities in children under 15 months of age. All of the children in this study that had ICH identified on CT/POCUS brain were admitted to hospital for observation, thus meeting the PECARN definition of clinically significant injury. None to our knowledge required neurosurgical intervention. The most common indication for obtaining a scan where a patient had a positive brain POCUS result was trauma in assessing for intracranial hemorrhage (Figure 1, Figure 2). Additionally, the most common non-fracture positive result on CT was intracranial hemorrhage. This study did not look at skull fracture on POCUS as this has been previously studied and has shown to have good sensitivity 8. Although still a rare event, we had a relatively high percentage of patients with a positive CT for hemorrhage and of those, brain POCUS was positive in most cases. Two patients had a positive CT scan but a negative brain POCUS. The first patient had a small occipital extra-axial hemorrhage, as reported by radiology that was not seen on POCUS brain. The second patient had a small subdural in the parietal region. These cases highlight some of the limitations of brain POCUS when assessing the convexities of the head, which are hidden due to boundaries in the sonographic window. None of the extra-axial hemorrhages had a mass effect over the adjacent brain parenchyma. None of the patients that had a false-negative brain POCUS underwent surgical intervention. All patients with intracranial hemorrhage were admitted to our hospital for observation by the neurosurgical team. Most infants with head trauma that have not returned to baseline (more irritable, tired, poor feeding etc.) will be admitted to hospital regardless of imaging findings for observation as is our protocol at our institution.

The main barrier to routine use of brain POCUS in the ED is the patency of the anterior fontanelle, which serves as the necessary acoustic window. The anterior fontanelle is patent in more than 75% of infants at 12 months with a steady decline in patency over the next year and only 10% of infants having a patent fontanel by the age of 2 9. Furthermore the view through the anterior fontanelle alone limits views of the infratentorial structures including the cerebellum 10. This is overcome by using the mastoid fossa as a second acoustic window to examine fully the brain of the infant/neonate. 10, 11. Despite this view being used in the neonatal literature, we did not detect any abnormalities with this view, and in most children >4 weeks old, the opacification of the mastoid limited anatomical views significantly. 

When developing this study, we decided to have a radiologist over-read all our brain POCUS images. We anticipated that the radiologist might be able to pick up subtle findings on brain POCUS that may not have been diagnosed by the clinician. Although the radiologist found the images obtained were of sufficient quality, he was not able to see all the positive findings attained by the POCUS brain clinician. This may have been for several reasons, but is likely because POCUS is dynamic, and the clinician cannot be blinded to the patient’s clinical status (e.g., knowing where the injury is on the head, may help to focus on where to look for findings on POCUS). They may be in a better position to read the images than someone not involved in the care who only has select clips or images. As a result, the inter-rater reliability was lower than anticipated, but arguably could have been predicted due to different clinical circumstances between the ER physician and radiologist. This further highlights the added benefit of POCUS; it allows one individual to do multiple tasks that normally depend on many specialists. This increases efficiency and may improve workflow. 

Literature on the use of POCUS to identify intracranial anomalies is limited despite emergency room physicians expanding its use. A recent study by Subramaniam et. al. reviewed the use and technique of transfontanellar sonography in the emergency department to identify hydrocephalus and highlights its importance as an easy to learn technique as well as a quick and accurate modality 12. Several other studies have retrospectively looked at POCUS of the brain and compared it to the findings on CT. Elkhunovich et. Al. completed their study reviewing the sensitivity and specificity of POCUS brain in conjunction with CT or MRI done for infants presenting to hospital with suspected intra-cranial hemorrhage 3. Although this study was performed retrospectively and POCUS brain was not being done with the direct purpose of looking acutely for intracranial pathology, they found a similar sensitivity at 67% and specificity of 99% 3. In a non-blinded study, McCormick et. al performed a prospective study where POCUS was used after positive CT showed evidence of hemorrhage 6. This study only had 12 patients, 4 of whom had CT imaging done for the direct purpose of evaluating for ICH. 

To our knowledge, our study is the first to prospectively perform brain POCUS and compare it to CT on patients presenting to the ED. This study is the only study to capture the use of POCUS for the brain used in the same context that other ED POCUS modalities are used for: to attain additional information about a patient that can help determine diagnosis or disposition. We limited our brain POCUS scans to be done within a 2-hour time frame on either side of the CT scan to not favourably bias brain POCUS if a bleed progressed and therefore would have been easier to see several hours later. Further, we had PEM POCUS trained staff performing the brain POCUS in the clinical setting which is how POCUS is used every day in the ED. Unlike CT, POCUS brain can be repeated as many times as necessary to further evaluate the patient if there is a clinical need. This may be useful, particularly in PECARN intermediate risk patients who require a period of observation. As both CT and POCUS brain can be falsely negative if performed too early after a bleed, this would be a safe and efficient way to repeat imaging over time. 

The limitations of our study include that most of the brain POCUS scans were performed by the primary investigator (SD). We also had a very high CT positivity rate which is much higher than the published positivity rates of 3-5% 3 and this may have led to selection bias. Halfway through our study our department upgraded our POCUS machine resulting in much better-quality images. This may have affected the false negative rate in the first year of this study and may speak to the potential that newer machines, with better technology, could improve the results of studies like ours. Lastly, there were no positive findings in our study that were picked up in the mastoid view. This could be from lack of experience using this acoustic window, or decreased sensitivity of this view in our patient population. Further studies may be required to further elucidate this difference. 

## Conclusion

This study shows that brain POCUS is an imaging modality with reasonable sensitivity and specificity in identifying intracranial pathologies that are present on CT in the ED setting. Its use would be most beneficial to expedite definitive imaging and subspecialty involvement, and for patients that have had a clinical change during a period of observation. For example, an infant that presents with decreased level of consciousness in an ED in whom a bleed is picked up on brain POCUS could have the neurosurgical and intensive-care physicians notified of their status while awaiting CT which could potentially expedite definitive management. Future research should be aimed at multicentre studies that could further elucidate the precision of POCUS brain in the setting of young infants presenting to the ED.

## Disclosures

The authors have no conflicts of interest to disclose. All funding was awarded through the Research Institute at the Children’s Hospital of Eastern Ontario, Fellow Research Grant.

## Patient Consent

Patient consent was obtained by the authors in addition to approval from the hospital ethics department.

## Supplementary Material 

Supplementary Appendix 1 Emergency department point of care head ultrasound in children less than 15 months of age receiving computed tomography: A proof of concept study.

## References

[R214214329518727] Marin J R, Abo A M, Arroyo A C, Doniger S J, Fischer J W, Rempell R (2016). Pediatric emergency medicine point-of-care ultrasound: summary of the evidence. Crit Ultrasound J.

[R214214329518731] Marin J R, Lewiss R E, Shook J E, Ackerman A D, Chun T H, Conners G P (2015). Point-of-care ultrasonography by Pediatric Emergency medicine physicians. Pediatrics.

[R214214329518728] Elkhunovich M, Sirody J, Mccormick T, Goodarzian F, Claudius I (2018). The utility of cranial ultrasound for detection of intracranial hemorrhage in infants. Pediatric Emergency Care.

[R214214329518732] Halm B M, Franke A (2011). Diagnosis of an intraventricular hemorrhage by a pediatric emergency medicine attending using point-of-care ultrasound: a case report. Pediatr Emerg Care.

[R214214329518721] Adcock L M, Moore P J, Schlesinger A E, Armstrong D L (1998). Correlation of ultrasound with postmortem neuropathologic studies in neonates. Pediatr Neurol.

[R214214329518729] Mccormick T, Chilstrom M, Childs J, Mcgarry R, Seif D, Mailhot T (2015). Point-of-Care Ultrasound for the Detection of Traumatic Intracranial Hemorrhage in Infants: A Pilot Study. Pediatr Emerg Care [Internet].

[R214214329518724] Bressan S, Eapen N, Phillips N, Gilhotra Y, Kochar A, Dalton S (2021). PECARN algorithms for minor head trauma: Risk stratification estimates from a prospective PREDICT cohort study. Acad Emerg Med.

[R214214329518723] Alexandridis G, Verschuuren E W, Rosendaal A V, Kanhai D A (2020). Evidence base for point-of-care ultrasound (POCUS) for diagnosis of skull fractures in children: A systematic review and meta-analysis. Emerg Med J.

[R214214329518725] Pindrik J, Ye X B, Ji B G, Pendleton C, Ahn E S (2014). Anterior Fontanelle Closure and Size in Full-Term Children Based on Head Computed Tomography. Clin Pediatr (Phila).

[R214214329518722] Steggerda S J, Bruïne F T De, Smits-Wintjens Vehj, Walther F J, Wezel-Meijler G Van (2012). Ultrasound detection of posterior fossa abnormalities in full-term neonates. Early Hum Dev.

[R214214329518730] Parodi A, Rossi A, Severino M, Morana G, Sannia A, Calevo M G (2015). Accuracy of ultrasound in assessing cerebellar haemorrhages in very low birthweight babies.. Arch Dis Child - Fetal Neonatal Ed.

[R214214329518726] Subramaniam S, Chen A E, Khwaja A, Rempell R (2019). Identifying infant hydrocephalus in the emergency department with transfontanellar POCUS. Am J Emerg Med.

