# Supplementary material for: Brain Point of Care Ultrasound in Young Children Receiving Computed Tomography in the Emergency Department: A Proof of Concept Study
Source: POCUS J. 2023 Nov 27;8(2):165–9. doi: 10.24908/pocus.v8i2.16435 (PMC10721297; doi:10.24908/pocus.v8i2.16435)
Supplement: Supplementary Appendix 1 [file pocusj-08-16435-s001.pdf]

Emergency department point of care head ultrasound in children less than 15 months of age receiving computed tomography: A proof of concept study.

POCUS BRAIN TRAINING

A. The study team included PEM fellows and selected PEM staff who were each trained during a 60-min seminar developed by the PI and co-author from the NICU (NBF), outlining the principles of brain ultrasound in a point-of-care setting. This seminar focused on identifying the major landmarks of the neonatal and infant brain and recognizing normal versus abnormal findings.

B. The workshop lasted around 60-90 mins. We anticipated this would be sufficient for learning the basics for screening. Multiple studies have shown that pediatric emergency physicians with prior POCUS experience can attain reasonable competency with brief (<2h) educational interventions in a wide array of diagnosis including fractures, appendicitis, pyloric stenosis and skin infections[1234]

C. All the PEM fellows and staff participating in this study received a POCUS introductory course and clinical practice prior to being selected for this study.

D. The PEM fellows and staff were given the opportunity to practice POCUS Brain on real infants during the workshop and on patients, who gave permission during “scanning” shifts in the ED.

References:

1. Barata I, Spencer R, Suppiah A. Emergency Ultrasound in the Detection of Pediatric Long-Bone Fractures. … *Emerg care*. 2012;28(11):1154-1157. http://journals.lww.com/pec-online/Abstract/2012/11000/Emergency_Ultrasound_in_the_Detection_of_Pediatric.8.aspx.
2. Marin JR, Dean AJ, Bilker WB, Panebianco NL, Brown NJ, Alpern ER. Emergency ultrasound-assisted examination of skin and soft tissue infections in the pediatric emergency department. *Acad Emerg Med*. 2013;20(6):545-553. doi:10.1111/acem.12148
3. Riera A, Chen L. Ultrasound evaluation of skull fractures in children: a feasibility study. *Pediatr Emerg Care*. 2012;28(5):420-425. doi:10.1097/PEC.0b013e318252da3b
4. Sivitz AB, Tejani C, Cohen SG. Evaluation of hypertrophic pyloric stenosis by pediatric emergency physician sonography. *Acad Emerg Med*. 2013;20(7):646-651. doi:10.1111/acem.12163
